# Dentists’ Knowledge, Attitudes, and Practices Regarding Molar Incisor Hypomineralization (MIH): A French Survey

**DOI:** 10.7759/cureus.78943

**Published:** 2025-02-13

**Authors:** Thomas Marquillier, Valérie Szönyi, Julia Mwenge-Wambel, Elisabeth Dursun, Brigitte Grosgogeat

**Affiliations:** 1 Pediatric Dentistry, Lille University Hospital Center, Lille, FRA; 2 Pediatric Dentistry, University of Lille, Lille, FRA; 3 Research, Sorbonne Paris North University, Education and Health Practices Laboratory (LEPS) Research Unit 3412, Bobigny, FRA; 4 Public Health, University Claude Bernard Lyon 1, Lyon, FRA; 5 Dental Consultation and Treatment Services, Lyon University Hospital Centre (HCL), Lyon, FRA; 6 Center for Epidemiology and Population Health Research (team BIOETHICS), French National Institute of Health and Medical Studies (INSERM) Mixed Research Unit 1295, Faculty of Health, University Toulouse III - Paul Sabatier, Toulouse, FRA; 7 Research, French Dental Practice-Based Research Network (ReCOL), Paris, FRA; 8 Pediatric Dentistry, Paris Cité University, Montrouge, FRA; 9 Pediatric Dentistry, Henri Mondor Hospital, Créteil, FRA; 10 Research, Innovative Dental Materials and Interface Research Unit, Montrouge, FRA; 11 Research, University Claude Bernard Lyon 1, Lyon, FRA; 12 Laboratory of Multimaterials and Interfaces (LMI), National Center for Scientific Research (CNRS) Mixed Research Unit 5616, Lyon 1 University, Faculty of Odontology, Lyon, FRA

**Keywords:** attitude, beliefs, dentists, knowledge, molar-incisor hypomineralisation, questionnaire

## Abstract

Introduction: With an estimated 878 million cases worldwide in 2015, molar incisor hypomineralization (MIH) is a growing issue. Early detection and management require knowledge and application of good clinical practices. The aim of the study was to evaluate the knowledge, attitudes, and practices of French dentists regarding MIH.

Method: A self-administered questionnaire was distributed to different groups of practitioners between 30 January and 27 March 2023, including members of the French dental practice-based research network, members of the social network “French pediatric dentists,” and members of the professional social network LinkedIn (LinkedIn, Sunnyvale, California, United States). The data from 311 questionnaires were analyzed.

Results: Around 277 (89%) practitioners were familiar with MIH, 180 (58%) were perfectly aware of the clinical features of MIH, and 193 (62%) reported being able to clinically identify MIH. Nearly 100% (310) of dentists cited “environmental pollutants” as the most frequent etiological factor. Glass ionomer is the most used restorative material.

Conclusion: Education regarding MIH must be improved among dentists to correctly detect, treat, or refer patients. It would seem useful to carry out an MIH prevalence study in France.

## Introduction

Molar incisor hypomineralization (MIH) is a growing concern worldwide. The number of cases in daily practice seems to have increased in recent years, requiring dentists to adapt their practices to address this issue [1]. The term MIH was proposed in 2001 [2], then adopted by the European Academy of Paediatric Dentistry (EAPD), and is now used worldwide [3,4]. MIH is a qualitative enamel defect of systemic origin, affecting one to four of the first permanent molars, frequently associated (in 70% of cases) with damage to the permanent incisors [2,3], and more rarely to the permanent second molars and canines [5]. Similar defects in primary teeth are also observed, known as hypomineralized second primary molars (HSPM) [6]. Clinically, these defects are characterized by demarcated opacities of various colors and extents, which can frequently undergo post-eruptive breakdown [3]. According to the degree of severity, the hardness of the hypomineralized enamel is, on average, six times less than that of healthy enamel [7]. The lack of protection of the dentin-pulp organ by a proper enamel barrier leads to many complications such as susceptibility to dental caries, hypersensitivity, tissue destruction, and chronic pulp inflammation. The pain, the unsightly incisors, and the difficult and repeated care sessions can impact the quality of life [8]. As such, the daily management of MIH represents a real challenge [9].

In France, the prevalence of MIH is unknown. The Global Burden of Disease study estimated that there were 878 million cases of MIH worldwide in 2015 [10]. According to a meta-analysis carried out in 2021 on 113,089 participants, the global prevalence of MIH was estimated at 13.5% (95% CI 12.1-15.1, p < 0.001) [11]. In Europe, it was estimated at 14.3% based on pooled data from 30 studies published between 1987 and 2016 and focusing on the four to 17-year age group [12].

The etiology of MIH is still unknown, but its multifactorial aspect is now well established. A possible genetic predisposition associated with disturbances during amelogenesis, and more specifically in the last stage of amelogenesis, has been suggested [13,14]. Given the chronology of teeth mineralization and the location of opacities, MIH represents the after-effects of events that occurred during pregnancy or early childhood (up to five years of age). The most sensitive period appears to be during the first year of life. Several etiological hypotheses regarding environmental factors have also been put forward (pathology, medication, stress, smoking, environmental toxins, diet, etc.) [14]. As the amelogenesis of primary molars takes place between the 15th week of life in utero and the first year of the child's life, HSPM is predictive of MIH in 20% of cases [6].

The management of MIH presents significant challenges due to several key factors, including accurate diagnosis, the necessity for early prevention, and the complex decisions surrounding the selection of restorative materials and techniques. By adopting an evidence-based approach, dentists can provide personalized and effective care for patients with MIH. This involves implementing specific preventive protocols, utilizing appropriate restorative materials, and conducting regular monitoring and long-term evaluation of results. Employing proven techniques and materials can minimize complications and enhance the quality of life for affected patients. Therefore, the knowledge, attitudes, and practices of dentists are crucial for generating, applying, and improving evidence. By staying informed about scientific advancements, fostering a supportive attitude toward research, and integrating evidence-based practices into their daily routines, dentists can contribute to the progression of evidence in the field and ensure high-quality care for their patients [15].

Although a number of studies have been carried out to assess the knowledge and attitudes of dentists regarding MIH in other countries such as Portugal, Spain, Norway, Australia, Chile, Indonesia, Syria, and Kuwait [16-23], only one local study has been carried out in France, targeting only the first permanent molar [24]. The aim of this study was thus to assess the knowledge, attitudes, and practices of French dentists regarding MIH, using an online questionnaire. The goal of this national study is to provide French data for a future epidemiological study with a higher level of evidence.

## Materials and methods

General considerations

The study lasted for one year, including the phases of conception, online dissemination, analysis of results, and the writing of the article. Given that the collaborators were not all based in the same city, communication was facilitated through video conferencing and email exchanges.

The electronic survey was conducted online [25]. In order to guarantee its methodological quality, the Checklist for Reporting Results of Internet E-Surveys (CHERRIES) recommendations were followed and explained (Table 1).

**Table 1:** Explanation of the CHERRIES recommendations. All the CHERRIES recommendations are described, including the study design, the information and consent process, the development and pre-testing of the survey questionnaire, the recruitment process and the description of the sample, the administration of the survey questionnaire, the response rates, the prevention of multiple entries from the same person, and the data relating to the analysis [25]. CHERRIES: Checklist for Reporting Results of Internet E-Surveys; IP: Internet Protocol

Item Category	Checklist Item	Explanation
Design	Describe survey design	This was a convenience sample. The target population was represented by different groups of practitioners, members of the French dental practice-based research network, members of the social network “Pédodontistes de France”, or members of the professional social network LinkedIn.
Institutional Review Board (IRB) approval and informed consent process	IRB approval	According to French regulations (Articles L1121-1 to L1121-3 of the French Public Health Code), ethics approval is required for research involving human participants only when sensitive or identifiable data are collected or when the study involves biomedical risks. This study did not involve the collection of sensitive, identifiable, or personal data, nor did it pose any risk to participants. As such, approval from an Institutional Review Board (IRB) was not required. Furthermore, all participants provided informed consent before participating, and data collection was anonymous and voluntary. The design of the study was approved by the French national authorities regulating confidentiality (CNIL, Commission Nationale Informatique et Libertés, No 2232858 v 0).
	Informed consent	Participants were informed of the purpose of the survey and of the time required to complete the questionnaire (less than 10 minutes). By clicking to access the study, the participants expressed their consent before completing the questionnaire.
	Data protection	No personal or sensitive data was collected as part of this survey. Only the respondent's year of birth was requested anonymously and without the possibility of identification. The data were collected on the SurveyMonkey platform (which complies with data protection laws) during the data collection period, then exported and stored on the principal investigator's (TM) password-protected computer.
Development and pre-testing	Development and testing	The questionnaire used in the survey was adapted from the literature (Bekes 2021). It was developed on a web-based platform and was pilot-tested on a sample of five dentists. Minor corrections and additions were made to the final survey after pilot testing.
Recruitment process and description of the sample having access to the questionnaire	Open survey versus closed survey	This was an open survey. Eligibility was assessed within the opening pages of the survey.
	Contact mode	Initial contact with participants was made via the Internet (e-mail or social networks).
	Advertising the survey	The survey was announced using online media (e-mail and social networks). The announcement is attached (Appendix 1).
Survey administration	Web/e-mail	The electronic survey was conducted on a website (SurveyMonkey), which enabled responses to be captured automatically. The website's link was provided directly to respondents via email or social networks.
	Context	SurveyMonkey is a free online survey platform that enables data to be collected and analyzed in accordance with data protection legislation. This site is generally consulted by invitation (it is a platform only).
	Mandatory/voluntary	It was a voluntary survey.
	Incentives	No incentive was offered to complete the survey.
	Time/Date	The data was collected between 30 January 2023 and 27 March 2023.
	Randomization of items or questionnaires	As the questionnaire called for logical responses, none of the items were randomized.
	Adaptive questioning	The adaptive questionnaire was not used.
	Number of Items	The survey consisted of 24 questions.
	Number of screens (pages)	The questionnaire was in one part (one page) without conditional connections. Sub-sections were formalized (introduction, general questions, two parts). No page switching was necessary. The number of screens was dependent on the type of device used to answer the questionnaire.
	Completeness check	The consistency (Alpha Cronbach) of the questionnaire was not checked. All questions included a non-response option such as "not applicable" or "rather not say", and the selection of an answer option was mandatory.
	Review step	Respondents could review their answers using the "previous" button.
Response rates	Unique site visitor	Having provided participation rates, unique visitors were identified by their IP address.
	View rate (ratio of unique survey visitors/unique site visitors)	N/A as participation was voluntary and respondents accessed the survey online via a dedicated link.
	Participation rate (ratio of unique visitors who agreed to participate/unique first survey page visitors)	Given the distribution method chosen for the survey, it was not possible to calculate this ratio.
	Completion rate (ratio of users who finished the survey/users who agreed to participate)	The completion rate was 100%.
Preventing multiple entries from the same individual	Cookies used	Cookies were used to ensure that a person could only complete the survey once.
	IP check	The IP address was recorded as metadata for the duration of the survey. If a person tried to complete the survey again from the same browser, a message was displayed informing him that they had already completed the survey.
	Log file analysis	No other technique was used.
	Registration	This was an open survey. A login was not used - entry to the survey was via a web link shared with eligible participants.
Analysis	Handling of incomplete questionnaires	Respondents were required to finish the questionnaire in order to validate their answers, thus no incomplete questionnaire could be considered.
	Questionnaires submitted with an atypical timestamp	No respondent was removed from the survey for completing the items with an atypical timestamp.
	Statistical correction	No statistical correction was performed.

Population

The target population consisted of dentists in private practices or employees of an organization (hospital, clinic, etc.) all over France, who were members of the French Dental Practice Research Network, and/or members of the “French pediatric dentists” social network, and/or members of the LinkedIn (LinkedIn, Sunnyvale, California, United States) professional social network.

Development of the survey questionnaire

The proposed questionnaire was adapted from that of Bekes et al. (2021) [26] and was developed using the SurveyMonkey website (https://uk.surveymonkey.com/). The adaptations to the questionnaire were minimal (a few rewordings). An explanatory text briefly described the context of the study and its ethical considerations. The questionnaire comprised 24 questions addressing the demographic characteristics of the population surveyed as well as their knowledge, attitudes, and practices relating to MIH and its management. All questions were multiple-choice questions, and the answering time was estimated to be less than 10 minutes. The questionnaire was tested between 10 January 2023 and 25 January 2023. It was first tested by five researchers, who did not express any wish for modification. The questionnaire was then tested by five practitioners. Only two of them indicated the need to change the order of the questions for greater consistency.

Questionnaire distribution

Any dentist interested in taking part in surveys or research activities in France can join the French dental practice-based research network (ReCOL). The network therefore sent the questionnaire to all its member practitioners via their mailing list (Appendix 1). We used an empirical sample, which is compatible with a descriptive analysis of the data. The questionnaire was sent on 31 January 2023, and a reminder was sent on 21 February 2023 as part of a thesis work. In addition, the questionnaire was distributed on social networks to practitioners in the “French pediatric dentists” group (Facebook, Meta Platforms, Inc., Menlo Park, California, United States) and via LinkedIn. The purpose of the questionnaire was described in the introduction to the e-mail sent out (or on the social network post), and a hypertext link was provided to access it; the practitioner completed the questionnaire online and validated his or her answers.

Receipt of replies

After being answered by a dentist online, responses were automatically recorded online. Responses were received between 30 January 2023 and 27 March 2023.

Statistical analysis

The results are presented using descriptive statistical analysis produced with EXCEL for Mac (16.78.3, Apple Inc., California, United States). Discontinuous variables were analyzed using their counts and percentages, while continuous variables were analyzed using their mean, standard deviation, minimum, and maximum values.

## Results

Characteristics of the respondents

Among the dentists invited to participate, 311 replied: 88 pediatric dentists (28.3%), 103 kid-friendly general dentists (33.1%), and 120 general dentists and other specialist dentists (such as orthodontists, 24 (7.9%)). Among the cases, 230 individuals, representing 73.9%, were female, and the mean (SD) age was 39.8 (9.5) years old; the mean age of pediatric dentists was lower (36.8 years), while that of the general dentists was higher (42.3 years). The respondents were from all French regions except “Corse”; 225 (82.0%) worked in a private practice, 69 (78.4%) for pediatric dentists, 87 (84.4%) for kid-friendly general dentists, 24 (7.7%) were employed in a private organization, and 25 (8.1%) were employed in a public hospital. Overall, 185 dentists (59.5%) were members of a learned society, 76 (41.1%) of whom belonged to ReCOL. Regarding pediatric dentists, 46 (71.9%) belonged to the French Society of Pediatric Dentistry (Table 2).

**Table 2:** Respondents’ characteristics, knowledge, attitudes, and practices regarding MIH. The table presents the data analyzed for the entire sample and according to three sub-groups: pediatric dentists, general dentists, and kid-friendly general dentists. *The weighted score is considered in order of importance from 1 to 10 and the results are described as the weighted score (number of respondents). **The weighted score is considered in order of importance from 1 to 11 and the results are described as the weighted score (number of respondents). ***The weighted score is considered in order of importance from 1 to 9 and the results are described as the weighted score (number of respondents). MIH: molar incisor hypomineralization; CAD: computer-aided design; CAM: computer-aided manufacturing; ODF: orthodontics; SFOP: French Society of Pediatric Dentistry; ReCOL: French Dental practice-based research network

	Question	Total Response rate N (%)	Pediatric dentist N (%)	General dentist (N (%)	"Kid-friendly" general dentist N (%)
1	Are you a man, a woman, or other?	311 (100)	88 (28.3)	120 (38.6)	103 (33.1)
	Man	81 (26.1)	9 (10.2)	45 (37.5)	27 (26.2)
	Woman	230 (73.9)	79 (89.8)	75 (62.5)	76 (73.8)
	Other	0 (0.0)	0 (0.0)	0 (0.0)	0 (0.0)
2	Year of birth?	311 (100)	88 (28.3)	120 (38.6)	103 (33.1)
	Age (years) Mean	39.8	36.8	42.3	39.5
	Age (years) Maximum	82	61.0	82.0	69.0
	Age (years) Minimum	25	26.0	25.0	25.0
	Age (years) Standard deviation	9.5	7.6	9.9	9.8
3	Where do you mainly practice?	308 (99.0)	87 (28.0)	120 (38.6)	101 (32.4)
	Auvergne-Rhône-Alpes (01, 03, 07, 15, 26, 38, 42, 43, 63, 69, 73, 74)	70 (22.7)	14 (16.1)	29 (24.2)	26 (25.7)
	Bourgogne-Franche-Comté (21, 25, 39, 58, 70, 71, 89, 90)	7 (2.3)	1 (1.2)	4 (3.3)	2 (1.9)
	Bretagne (22, 29, 35, 56)	18 (5.8)	3 (3.4)	8 (6.7)	7 (7.0)
	Centre-Val de Loire (18, 28, 36, 37, 41, 45)	6 (2.0)	1 (1.2)	1 (0.8)	4 (3.9)
	Corse (2A, 2B)	0 (0.0)	0 (0.0)	0 (0.0)	0 (0.0)
	Grand Est (08, 10, 51, 52, 54, 55, 57, 67, 68, 88)	22 (7.1)	4 (4.6)	10 (8.3)	8 (8.0)
	Hauts-de-France (02, 59, 60, 62, 80)	28 (9.1)	8 (9.2)	11 (9.2)	9 (9.0)
	Île-de-France (75, 77, 78, 91, 92, 93, 94, 95)	57 (18.5)	26 (29.9)	14 (11.7)	17 (16.8)
	Normandie (14, 27, 50, 61, 76)	10 (3.3)	3 (3.4)	6 (5.0)	1 (0.9)
	Nouvelle-Aquitaine (16, 17, 19, 23, 24, 33, 40, 47, 64, 79, 86, 87)	20 (6.5)	7 (8.1)	6 (5.0)	8 (8.0)
	Occitanie (09, 11, 12, 30, 31, 32, 34, 46, 48, 65, 66, 81, 82)	31 (10.1)	6 (6.9)	18 (15.0)	8 (8.0)
	Pays de la Loire (44, 49, 53, 72, 85)	13 (4.2)	3 (3.4)	7 (5.8)	2 (1.9)
	Provence-Alpes-Côte d'Azur (04, 05, 06, 13, 83, 84)	13 (4.2)	5 (5.7)	3 (2.5)	4 (3.9)
	Territoires ultra-marins : Guadeloupe, Guyane française, Martinique, La Réunion, Mayotte, Nouvelle-Calédonie, Polynésie française, Saint-Barthélemy, Saint-Martin, Saint-Pierre-et-Miquelon, Terres Australes et Antarctiques Françaises, îles de Wallis-et-Futuna (97, 98)	13 (4.2)	6 (6.9)	3 (2.5)	5 (5.0)
4	Your main mode of practice is	311 (100)	88 (28.3)	120 (38.6)	103 (33.1)
	Private practice	225 (82.0)	69 (78.4)	99 (82.5)	87 (84.4)
	An employee in a private organization	24 (7.7)	9 (10.2)	7 (5.8)	8 (7.8)
	Employed in a public hospital	25 (8.1)	6 (6.8)	12 (10.0)	7 (6.8)
	Other (please specify)	7 (2.2)	4 (4.6)	2 (1.7)	1 (1.0)
5	Do you specialize in pediatric dentistry?	311 (100)			
	Yes	88 (28.3)			
	No	120 (38.6)			
	No. But I receive a lot of children	103 (33.1)			
6	Do you have a specialist orthodontic practice?	304 (97 .7)	88 (28.3)	117 (37.6)	99 (31.8)
	Yes	24 (7.9)	4 (4.5)	8 (6.8)	12 (12.1)
	No	260 (85.6)	76 (86.4)	105 (89.7)	79 (79.8)
	No. But I perform orthodontic procedures	20 (6.5)	8 (9.1)	4 (3.5)	8 (8.1)
7	Do you belong to a learned society?	185 (59.5)	64 (20.6)	76 (24.4)	45 (14.5)
	ReCOL	76 (41.1)	7 (10.9)	47 (61.8)	22 (48.9)
	SFOP	51 (27.6)	46 (71.9)	0 (0.0)	5 (11.1)
	Other (please specify)	58 (31.3)	11 (17.2)	29 (38.2)	18 (40.0)
8	Are you familiar with MIH?	311 (100)	88 (28.3)	120 (38.6)	103 (33.1)
	Yes	277 (89.1)	87 (98.9)	97 (80.8)	93 (90.3)
	Moderately	34 (10.9)	1 (1.1)	23 (19.2)	10 (9.7)
	No	0 (0.0)	0 (0.0)	0 (0.0)	0 (0.0)
9	How did you hear about it? (one or more possible answers)	310 (99.7)	88 (28.3)	119 (38.3)	103 (33.1)
	Initial training	237 (76.4)	76 (86.4)	87 (73.1)	74 (71.8)
	Dental journals	205 (66.1)	58 (65.9)	71 (59.7)	76 (73.8)
	Conferences	174 (56.1)	61 (69.3)	56 (47.1)	57 (55.3)
	Internet	101 (32.6)	35 (39.8)	35 (29.4)	31 (30.1)
	Books	81 (26.1)	39 (44.3)	24 (20.2)	18 (17.5)
	Colleagues	96 (31.0)	35 (39.8)	33 (27.7)	28 (27.2)
10	Do you know the clinical features of MIH?	311 (100)	88 (28.3)	120 (38.6)	103 (33.1)
	Perfectly	180 (57.9)	80 (90.9)	41 (34.2)	59 (57.3)
	Moderately	128 (41.2)	8 (9.1)	77 (64.2)	43 (41.7)
	Not at all	3 (0.9)	0 (0.0)	2 (1.6)	1 (1)
11	Do you know if there are clinical criteria to diagnose MIH?	311 (100)	88 (28.3)	120 (38.6)	103 (33.1)
	Yes, and I know how to apply them	193 (62.1)	79 (89.8)	59 (49.2)	55 (53.4)
	Yes, but I do not know how to apply them	105 (33.7)	9 (10.2)	51 (42.5)	45 (43.7)
	No, I do not know	13 (4.2)	0 (0.0)	10 (8.3)	3 (2.9)
12	How confident do you feel when diagnosing MIH?	311 (100)	88 (28.3)	120 (38.6)	103 (33.1)
	Very confident	105 (33.8)	54 (61.4)	22 (18.3)	29 (28.2)
	Confident	161 (51.8)	31 (35.2)	68 (56.7)	62 (60.2)
	Slightly confident	37 (11.9)	3 (3.4)	23 (19.2)	11 (10.7)
	Not at all confident	8 (2.5)	0 (0.0)	7 (5.8)	1 (0.9)
13	Do you have difficulty distinguishing MIH from other developmental defects of enamel?	199 (64.0)	41 (13.2)	83 (26.7)	75 (24.1)
	Dental fluorosis	49 (24.6)	10 (24.4)	22 (26.5)	17 (22.7)
	Enamel hypoplasia	129 (64.8)	23 (56.1)	56 (67.5)	50 (66.7)
	Amelogenesis imperfecta	77 (38.7)	4 (9.8)	40 (48.2)	33 (44.0)
	Dentinogenesis imperfecta	26 (13.1)	0 (0.0)	16 (19.3)	10 (13.3)
	Trauma sequelae	40 (20.1)	8 (19.5)	14 (16.9)	18 (24.0)
	Other	13 (6.5)	7 (17.1)	5 (6.0)	1 (1.3)
14	Are you aware of the prevalence of MIH in Europe?	311 (100)	88 (28.3)	120 (38.6)	103 (33.1)
	Yes	86 (27.7)	46 (52.3)	13 (10.8)	27 (26.2)
	No	225 (72.3)	42 (47.7)	107 (89.2)	76 (73.8)
	If yes, could you indicate this, even approximately ()	92 (29.6)	45 (51.1)	18 (15.0)	29 (28.2)
	Mean prevalence	16.9	16.3	17.0	16.9
	Maximum prevalence	60.0	30.0	60.0	20.0
	Minimum prevalence	5.00	10.0	10.0	5.0
	Standard deviation	5.9	3.4	6.4	5.9
15	We do not yet know the prevalence of MIH in France. Do you think it would be useful to carry out a study?	310 (99.7)	87 (28.0)	120 (38.6)	103 (33.1)
	Yes	296 (95.5)	85 (97.7)	112 (93.3)	99 (96.1)
	No	14 (4.5)	2 (2.3)	8 (6.7)	4 (3.9)
16	How often do you notice these teeth in your clinical practice?	310 (99.7)	88 (28.3)	119 (38.3)	103 (33.1)
	Every month	184 (59.4)	35 (39.8)	71 (59.7)	78 (75.73)
	Yearly	45 (14.5)	1 (1.1)	36 (30.2)	8 (7.77)
	Other (specify)	81 (26.1)	52 (59.1)	12 (10.1)	17 (16.5)
	*Never or almost never	3 (3.7)	0 (0.0)	3 (25)	0 (0.0)
	*1 to a few times a year	7 (8.6)	0 (0.0)	4 (33.4)	3 (17.6)
	*1 to several times a month	3 (3.7)	1 (1.9)	1 (8.3)	1 (5.9)
	*1 to several times a week	45 (55.6)	31 (59.6)	3 (25.0)	11 (64.7)
	*1 to several times a day	23 (28.4)	20 (38.5)	1 (8.3)	2 (11.8)
17	In what proportion of patients do you observe MIH teeth?	311 (100)	88 (28.3)	120 (38.6)	103 (33.1)
	<10	137 (44.0)	8 (9.1)	79 (65.8)	50 (48.5)
	10-25	147 (47.3)	63 (71.6)	38 (31.7)	46 (44.7)
	If other (please specify)	27 (8.7)	17 (19.3)	3 (2.5)	7 (6.8)
	Mean proportion	33.7	35.9	41.3	35.39
	Maximum proportion	60.0	60	50.0	50
	Minimum proportion	2.0	26	32.5	2
	Standard deviation	13.8	9.9	10.2	11.9
18	Which of the following features do you most frequently notice regarding the severity of the defect? (one or more possible answers)	311 (100)	88 (28.3)	120 (38.6)	103 (33.1)
	White demarcation	120 (38.6)	39 (44.3)	50 (41.7)	31 (30.1)
	Yellow/brown demarcation	284 (91.3)	82 (93.2)	107 (89.2)	95 (92.2)
	Post-eruptive enamel breakdown	135 (43.4)	53 (60.2)	44 (36.7)	38 (36.9)
19	Have you encountered demarcated hypomineralized defects in permanent teeth other than the first permanent molars and incisors? Name the tooth/teeth	227 (73.0)	78 (25.1)	76 (24.4)	73 (23.5)
	Canines	112 (49.3)	47 (60.3)	34 (44.7)	31 (42.5)
	Premolars	115 (50.7)	47 (60.3)	30 (39.5)	38 (52.1)
	Second molars	112 (49.3)	43 (55.1)	34 (44.7)	35 (48.0)
20	How frequently do you notice demarcated hypomineralized lesions in the second primary molar tooth in comparison to the first permanent molar tooth?	309 (99.4)	87 (28.0)	119 (38.3)	103 (33.1)
	More frequently	21 (6.8)	12 (13.8)	6 (5.1)	3 (2.9)
	Less frequently	193 (62.5)	55 (63.2)	70 (58.8)	68 (66.0)
	The same as for the first permanent molar	30 (9.7)	14 (16.1)	8 (6.7)	8 (7.8)
	Never seen	65 (21.0)	6 (6.9)	35 (29.4)	24 (23.3)
21	Which factor(s) do you think are involved in the etiology of MIH? (All the possible answers must be ranked in order of importance, 1 being the most important. Please rank all the answers even if they do not seem relevant to you).*	311 (100)	88 (28.3)	120 (38.6)	103 (33.1)
	Genetic factors N (number of respondents)	6.8 (302)	6.2 (87)	7.0 (114)	7.1 (101)
	Chronic medical condition(s) of the mother during pregnancy	6.7 (300)	6.3 (86)	6.9 (114)	6.6 (100)
	Chronic medical condition(s) of the child concerned	6.8 (304)	7.1 (87)	6.5 (114)	6.8 (103)
	Antibiotics/medications taken by the mother during pregnancy	5.8 (299)	5.5 (86)	6.2 (113)	5.6 (100)
	Antibiotics/medications taken by the child concerned	5.8 (303)	6.4 (87)	5.7 (115)	5.3 (101)
	Environmental pollutants	8.1 (310)	7.9 (88)	8.0 (119)	8.4 (103)
	Acute medical condition(s) of the mother during pregnancy	5.5 (298)	5.6 (86)	5.4 (112)	5.6 (100)
	The acute medical condition(s) of the child concerned	5.3 (304)	6.2 (87)	4.9 (115)	5.0 (102)
	Exposure to fluoride	2.6 (300)	2.2 (86)	2.8 (113)	2.6 (101)
	None	2.0 (298)	1.6 (84)	1.9 (112)	2.4 (102)
22	Which material do you use MOST in treating MIH molars? (All the possible answers must be ranked in order of importance, 1 being the most important). Please rank all the answers even if they do not seem relevant to you). **	304 (97.8)	87 (28.0)	116 (37.3)	101 (32.5)
	Amalgam N (Number of respondents)	2.2 (291)	1.6 (83)	2.6 (111)	2.2 (97)
	Composite resin	8.8 (298)	8.8 (87)	8.9 (113)	8.7 (98)
	Fluid composite resin	7.7 (294)	7.5 (84)	8.1 (112)	7.4 (98)
	Glass ionomer cement	9.1 (301)	9.3 (85)	9.0 (115)	9.2 (101)
	Compomer	5.3 (294)	4.7 (83)	5.7 (112)	5.4 (99)
	Resin-modified glass ionomer cement	8.7 (299)	8.7 (84)	8.8 (114)	8.7 (101)
	Preformed crown	5.9 (298)	7.0 (87)	5.4 (111)	5.6 (100)
	Composite resin laboratory onlay	5.8 (297)	6.3 (84)	5.6 (113)	5.8 (100)
	Ceramic laboratory onlay	4.7 (296)	4.7 (83)	4.5 (112)	4.7 (101)
	CAD/CAM composite resin onlay	4.6 (294)	4.5 (83)	4.5 (112)	4.7 (99)
	CAD/CAM ceramic onlay	3.5 (295)	3.3 (83)	3.4 (112)	3.8 (100)
23	Which factors influence your choice of restorative material? (several answers possible, in order of importance, 1 being the most important). Please rank all the answers even if they do not seem relevant to you. ***	295 (94.9)	86 (27.7)	113 (36.3)	96 (30.9)
	Adhesion N (Number of respondents)	7.8 (293)	7.6 (85)	8.0 (113)	7.7 (95)
	Aesthetics	4.8 (288)	4.4 (84)	5.1 (11)	4.8 (93)
	Patient/parent preference	3.2 (289)	3.1 (84)	3.2 (110)	3.3 (95)
	Durability	6.5 (290)	6.5 (84)	6.8 (113)	6.3 (93)
	Remineralisation potential	6.5 (293)	6.6 (86)	6.4 (113)	6.6 (94)
	Sensitivity	5.7 (31)	5.5 (2)	5.7 (18)	5.8 (11)
	Personal experience	4.5 (290)	4.7 (85)	4.4 (111)	4.6 (94)
	Research findings	4.4 (287)	4.4 (84)	4.3 (110)	4.5 (93)
	Dental sensitivities	5.9 (292)	6.9 (86)	5.2 (110)	5.9 (96)
24	Do you think MIH poses a clinical challenge? If so, what is the most challenging? (one or more answers possible)	307 (98.7)	87 (28.0)	120 (38.6)	100 (32.1)
	Diagnosis N (number of respondents)	57 (18.6)	7 (8.1)	31 (25.8)	19 (19.0)
	Aesthetics	123 (40.1)	35 (40.2)	54 (45.0)	34 (34.0)
	Achieving adequate local anesthesia	164 (53.4)	57 (65.5)	57 (47.5)	50 (50.0)
	Determining the restorative margins of affected enamel	176 (57.3)	45 (51.7)	71 (59.2)	60 (60.0)
	Providing adequate restorations	163 (53.1)	38 (43.7)	70 (58.3)	55 (55.0)
	The long-term success of restorations	256 (83.4)	70 (80.5)	98 (81.7)	88 (88.0)
	Achieving patient comfort (for function, oral hygiene)	95 (30.9)	37 (42.5)	29 (24.2)	29 (29.0)
	Financial support	121 (39.4)	43 (49.4)	40 (33.3)	38 (38.0)
	If other (please specify)	13 (4.2)	6 (6.9)	3 (2.5)	4 (10.5)
	*Sensitivity	4 (30.8)	2 (33.3)	0 (0.0)	2 (50.0)
	*ODF bonding	2 (15.4)	0 (0.0)	1 (33.3)	1 (25.0)
	*Early diagnosis	1 (7.7)	1 (16.7)	0 (0.0)	0 (0.0)
	*Residual tissue	1 (7.7)	0 (0.0)	1 (33.3)	0 (0.0)
	*Cooperation	2 (15.3)	2 (33.3)	0 (0.0)	0 (0.0)
	*Clinical history	1 (7.7)	1 (16.7)	0 (0.0)	0 (0.0)
	*Etiology unknown	1 (7.7)	0 (0.0)	1 (33.34)	0 (0.0)
	*Isolation	1 (7.7)	0 (0.0)	0 (0.0)	1 (25.0)

Respondent's knowledge of molar incisor hypomineralization

Practitioners treating children were more familiar with MIH, with 87 pediatric dentists (98.9%) and 93 kid-friendly general dentists (90.3%), compared to 97 general dentists (80.8%).

No practitioner reported not being at all familiar with MIH. Overall, 237 respondents (76.4%) had been made aware of MIH during their initial training, 205 (66.1%) were confronted with the topic in dental journals, 174 (56.1%) in conferences, 101 (32.6%) on the internet, 81 (26.1%) in books, and 96 (31%) by colleagues. It should be noted that pediatric dentists were more familiar with MIH: 87 (98.9%) and had mainly heard of it during initial training (76 (86.4%)) (Table 2).

Among all respondents, 180 (57.9%) were perfectly familiar with the clinical features of MIH, and 193 (62.1%) knew how to apply its diagnostic criteria. Only 105 (33.8%) reported being very confident in diagnosing MIH, 54 (61.4%) of them being pediatric dentists, and 161 (51.8%) reported being confident. Moreover, 199 (64%) dentists answered having difficulties distinguishing MIH from other developmental defects, especially enamel hypoplasia: 129 (64.8%). While general and kid-friendly dentists reported having difficulties in distinguishing MIH from amelogenesis imperfecta (77 (38.7%) and 33 (44.0%), respectively) and dentinogenesis (26 (13.1%) and 10 (13.3%), respectively), none of the pediatric dentists reported having this issue for dentinogenesis and only four (9.8%) for amelogenesis imperfecta (Table 2).

Regarding etiology, 310 out of 311 dentists cited “environmental pollutants” as the most frequent etiological factor, followed by “chronic medical condition(s) of the child concerned” and “acute medical condition(s) of the child concerned.” Only 86 (27.7%) of all respondents, but 46 pediatric dentists (52.3%), replied that they were aware of the prevalence of MIH in Europe; 296 (95.5%) answered that setting up a study on this topic would be useful (Table 2).

Respondents’ clinical experience of molar incisor hypomineralization

A total of 137 (44%) of respondents reported having observed MIH in fewer than approximately 10% of their patients, and this was the case for 79 (65.8%) general dentists. For 147 (47.3%) respondents, MIH was observed in 10 to 25% of their patients, a proportion that was higher in pediatric dentists: 63 (71.6%). Yellow/brown demarcated opacities were the most common defects reported: 284 (91.3%). More pediatric dentists reported encountering post-eruptive enamel breakdown: 53 (60.2%) compared to non-specialist dentists (44 (36.7%) general dentists and 38 (36.9%) kid-friendly dentists). Overall, 227 (73.0%) dentists had encountered demarcated hypomineralized defects on permanent premolars, canines, and/or second molars, while 193 (62.5%) reported encountering less frequent lesions on the second primary molars (HSPM) than on the first permanent molars: 30 (9.7%).

Respondents’ practices for molar incisor hypomineralization management

When asked about the choice of material for restoration of MIH molars, out of the 304 respondents, 301 reported the use of glass ionomer cement (9.1%), 299 used resin-modified glass ionomer cement, and 297 used composite resin. It should be noted that the use of preformed crowns was more cited (298/304) than esthetic indirect alternatives represented by the different onlays. The factors most cited as influencing their choice of material were “adhesion” and “remineralization potential” (293 respondents). MIH was viewed as a clinical problem by 307 respondents (98.7%); among them, 256 (83.4%) viewed the long-term success of the restorations as challenging, while achieving adequate local anesthesia and providing adequate restorations was viewed as problematic for 164 (53.4%) and 163 (53.1%) of dentists, respectively.

## Discussion

The present survey highlighted that the knowledge, attitudes, and practices of French dentists regarding MIH differ according to their clinical practice. It is important to note, however, that this is a survey of practices, with limits that have to be kept in mind. When asked about MIH management, the results show that the EAPD recommendations are not necessarily followed [9] notably regarding the choice of material. The findings presented herein underline the need to further improve training and awareness of French dentists regarding the diagnosis and management of MIH.

Characteristics of the respondents

As in similar studies carried out in other countries [16,18-21], except the Syrian study [22], the majority of respondents herein were female. This may be explained by the fact that MIH is frequently treated during childhood and that women are still the most involved in the management of pediatric diseases [27]. The age of the respondents herein was also in line with the majority of studies reporting mean ages above 30 years old, except for studies carried out in Syria [22] and Portugal [16], where respondents were under 30 years old.

In terms of geographical representativeness, the respondents herein came from all French regions except one as well as from the overseas territories, which differs from the previous French survey, which was based in only one region [24]. This was enabled by the deployment method used for the online survey, which allowed reaching respondents without focusing on a single recruitment location.

The majority of the respondents worked in a private practice, which underlines the good representativeness of the present sample. However, almost a third of the respondents specialized in pediatric dentistry, a much higher proportion than the actual repartition of pediatric dentists in France. The online distribution of the questionnaire, notably on a social network where pediatric dentists are a majority, likely partly explains this repartition. Contrary to the previous French study, whose sample included almost 21% of orthodontists [24], orthodontists were poorly represented in the present sample. This is consistent with other studies [16-23], and could partly be due to the fact that MIH is primarily diagnosed and managed before the patient is referred to an orthodontist, making this population less targeted in studies dealing with MIH.

Respondent's knowledge of molar incisor hypomineralization

According to the present study, a large majority of dentists in France are familiar with MIH. This proportion seems to vary greatly between studies, as it was found to be higher in Australia, where the respondents were all members of the Society of Paediatric Dentistry [19], but more than twice as low in Portugal (41.2%), whereas only a minority of respondents were pediatric dentists [16]. This is in line with the present results showing that pediatric dentists are more familiar with MIH than general dentists, a finding previously reported [28]. All these studies, however, were not conducted at the same time, underlining a possible increase in knowledge regarding MIH over time. Interestingly, although almost all dentists reported knowing either moderately or perfectly well the clinical signs of MIH, only two-thirds declared that they knew how to apply the diagnostic criteria. It seems to support the fact that this apparent familiarity is not a guarantee of competence in diagnosing the condition. This gap was also observed in the Syrian study, which reported that 47% of respondents felt confident in diagnosing MIH, but only 26% knew the clinical diagnostic criteria and knew how to apply them [22]. If self-confidence in their ability to make the diagnosis is discordant with their knowledge of the clinical diagnostic criteria and their application, it is likely that many dentists underestimate the real number of MIH cases they encounter.

The main source of information about MIH reported herein was initial training courses, followed by dental journals, which is in line with the fact that MIH has been widely taught at universities in France for several years. This result seems to support the fact that similar findings show that training courses and dental journals appear to be the main sources of knowledge for MIH [18,21,22]. In a study carried out in Spain, however, the first source of information on MIH for dentists was the Internet, behind dental education [17]. While it makes sense that dental education and journals provide the primary source of information, internet-based learning appears important for young practitioners [29]. Nevertheless, after their initial training, it was through conferences that pediatric dentists had more frequently heard about MIH. This may be because pediatric dentists are more widely exposed to this condition, rendering MIH a very frequent subject for conferences.

The frequency with which dentists were confronted with MIH was rather low, as almost two-thirds of them reported seeing patients with MIH on a monthly basis. Since it is necessary to be regularly confronted with MIH in order to take an interest in it, be trained, and thus understand the specificities related to its diagnosis and management, this frequency could explain why respondents seemed only partially familiar with the condition.

Less than one-third of dentists knew the prevalence of MIH in Europe, yet although most studies on the prevalence of MIH have been conducted in European countries, only less than a third of dentists knew the prevalence of MIH in Europe. The respondents had difficulty in mentioning the prevalence of the disease, probably because of the lack of French epidemiological data, indicating that research may be biased toward diagnosis and treatment aspects rather than epidemiology.

Respondents’ clinical experience of molar incisor hypomineralization

In the present study, more than two-thirds of the general dentists reported a prevalence of approximately less than 10% in their patients, whereas three-quarters of the pediatric dentists reported a prevalence between 10 and 25%. If it is important to keep in mind the possible existence of a recall bias, this prevalence, which reflects the European prevalence, appears entirely coherent as most patients with MIH are referred to pediatric dentists, and suggests that the French prevalence of MIH could be between 10 and 25%. The prevalence of MIH reported by dentists appears to vary between countries, as dentists in Norway reported a similar trend (10 to 20%) [18], but 49% of dentists in Spain indicated observing MIH in less than 10% of patients [17], and 21% of dentists in Indonesia reported observing it in less than 5% of cases [21]. Such disparities are not surprising as epidemiology depends on a number of factors, such as the ability to make a diagnosis, the population affected, as well as the environment. In terms of etiology, genetics is thought to be a predominant factor, as reported by a majority of dentists in many studies [16,18,21], but environmental pollutants likely play an important role and were reported herein as the first factor by nearly all respondents [30].

Regarding the clinical signs related to the severity of the most observed defect, the large majority of the respondents herein indicated "yellow/brown demarcation," a result that was similar among pediatric and general dentists. This feature thus seems to be well identified among dentists during the diagnosis of MIH, which is in line with the data from the Spanish [17], for instance. Pediatric dentists encountered nearly twice as often post-eruptive enamel fractures than non-specialized dentists, probably because they received many patients referred lately in the course of the disease.

Moreover, three-quarters of the dentists herein reported having seen similar hypomineralized defects on premolars/canines/second molars. This is consistent with a similar study carried out among Australian and Chilean dentists [20] and coincides with a lower HSPM prevalence than MIH prevalence [31]. Respondents herein indicated that enamel hypoplasia was the main enamel defect that they could confuse with MIH. However, non-specialized dentists also had difficulties in distinguishing MIH from imperfecta amelogenesis and dentinogenesis, which was not the case for pediatric dentists. This underlines an important lack of knowledge, given the clinical differences between these various conditions.

Respondents’ practices for molar incisor hypomineralization management

Regarding the restorative materials used, glass ionomer cement came first in the present study, which is in agreement with the previous French study [24]. Yet, European recommendations suggest using glass ionomer cement as a temporary material, as its mechanical properties are inferior and less compatible with durable restorations in the posterior teeth [9]. The most used materials in other countries were resin composite in Portugal [16], resin-modified glass ionomer in Spain [17], resin composite and/or (resin-modified) glass ionomer in Australia and Chile [20], and preformed metal crowns or resin composite in Syria [22]. It has been suggested that resin composite should be preferred, as it has highly suitable properties and is easy to apply, despite salivary isolation being necessary. The preferred choice of glass ionomer cement by French dentists could be explained by the fact that most young patients with MIH are difficult to manage, anxious, and often fail to receive care. Dentists therefore prefer procedures that are simpler to perform, giving greater importance to the patient's well-being than to the biomechanical qualities of the materials used. This is contradictory to the fact that adhesion was reported as the primary factor influencing the choice of material. These findings, however, indicate a possible need to reevaluate current recommendations, which should also consider the constraints related to management protocols rather than the biochemical properties of restoration material alone. This is further supported by the results obtained in Norway and Kuwait, where the behavior of the child was viewed as the main challenge [18,23].

Strengths and limitations of the study

One of the strengths of this study is the large number of respondents and their repartition across the entire country, with different types of practices represented (private practice was predominant, as is the case for dentistry in France). In addition, the study was not conducted exclusively among pediatric dentists but in a varied sample. The equivalent proportions of pediatric dentists, general dentists, and kid-friendly dentists allowed comparisons to be made according to the type of practice. These different elements contribute to the representativeness of the study population. To limit recruitment bias, we targeted professional mailing lists (with secure emails) and professional networks (closed, requiring identification). These strengths make it possible to have a more nuanced view, closer to the actual practice in the field. This provides a starting point for a larger-scale study that could explore certain aspects more specifically. This type of study makes it possible to raise awareness within a professional network of the value of participating in research, and this is necessary in the case of studies carried out in private practice. It also enables us to focus more precisely on the questions for future studies: in our case, investigating the use of glass ionomer rather than composite, for example, which goes against the recommendations.

Although adapting a questionnaire previously validated and used in several studies allowed us to compare results across countries with relative reliability, it prevented us from designing a questionnaire tailored to more current considerations and the specificities of the French population of dentists. Lastly, the electronic distribution through mail and social networks may have led to a selection bias by only reaching practitioners comfortable with these communication channels.

## Conclusions

Education regarding MIH must be improved among dentists to correctly detect, treat, or refer patients. This is particularly true for general dentists. Moreover, it would seem relevant to carry out a prevalence study in France in order to specify the type and severity of the affected teeth. Since this is a national study, the results could guide a future study with a higher level of evidence.

## Appendices

Appendix 1 

**Table 3:** Messages accompanying the questionnaire distribution.

Messages accompanying the questionnaire distribution
First message accompanying the e-mail distribution
Dear ..., January is now well under way. We are back online to wish you all the best for 2023 and to tell you that ReCOL already has a number of projects that we will be sharing with you over the coming months. To begin with, we would be delighted if you would take part in a survey aimed at advancing knowledge of molar-incisor hypomineralization (MIH). All you need to do is take 5 minutes and click on the following link. Thank you very much for your contribution, and we will be sure to get back to you with the results. For the record, you can find ReCOL on its website and social networks. See you soon, The ReCOL team
Second message accompanying the e-mail distribution (when the reminder was sent)
Dear Members, You are wonderful! There have already been more than 150 responses to the MIH survey. Thank you to all of you who have already taken 5 minutes to reply. For all the others, it is not too late to make your contribution too. Just click on the following link. Thank you very much and see you soon. The ReCOL team
Message accompanying the distribution on social networks
Dear friend, You are reading this email because you are a valuable contributor to us and we thank you for that. Our MIH survey is coming to an end... but it hasn't reached its goal yet. We won't get any further without you! The aim of our survey is to assess your knowledge and attitudes when, every day in your practice, you are confronted with MIH. Whether you are a beginner or an experienced practitioner, give us 5 precious minutes of your time to help us advance research and design tomorrow's projects. We are counting on you!
